# Supplementary figures and images for: Fecal Metagenomics Study Reveals That a Low-Fiber Diet Drives the Migration of Wild Asian Elephants in Xishuangbanna, China
Source: Animals (Basel). 2023 Oct 13;13(20):3193. doi: 10.3390/ani13203193 (PMC10603651; doi:10.3390/ani13203193)

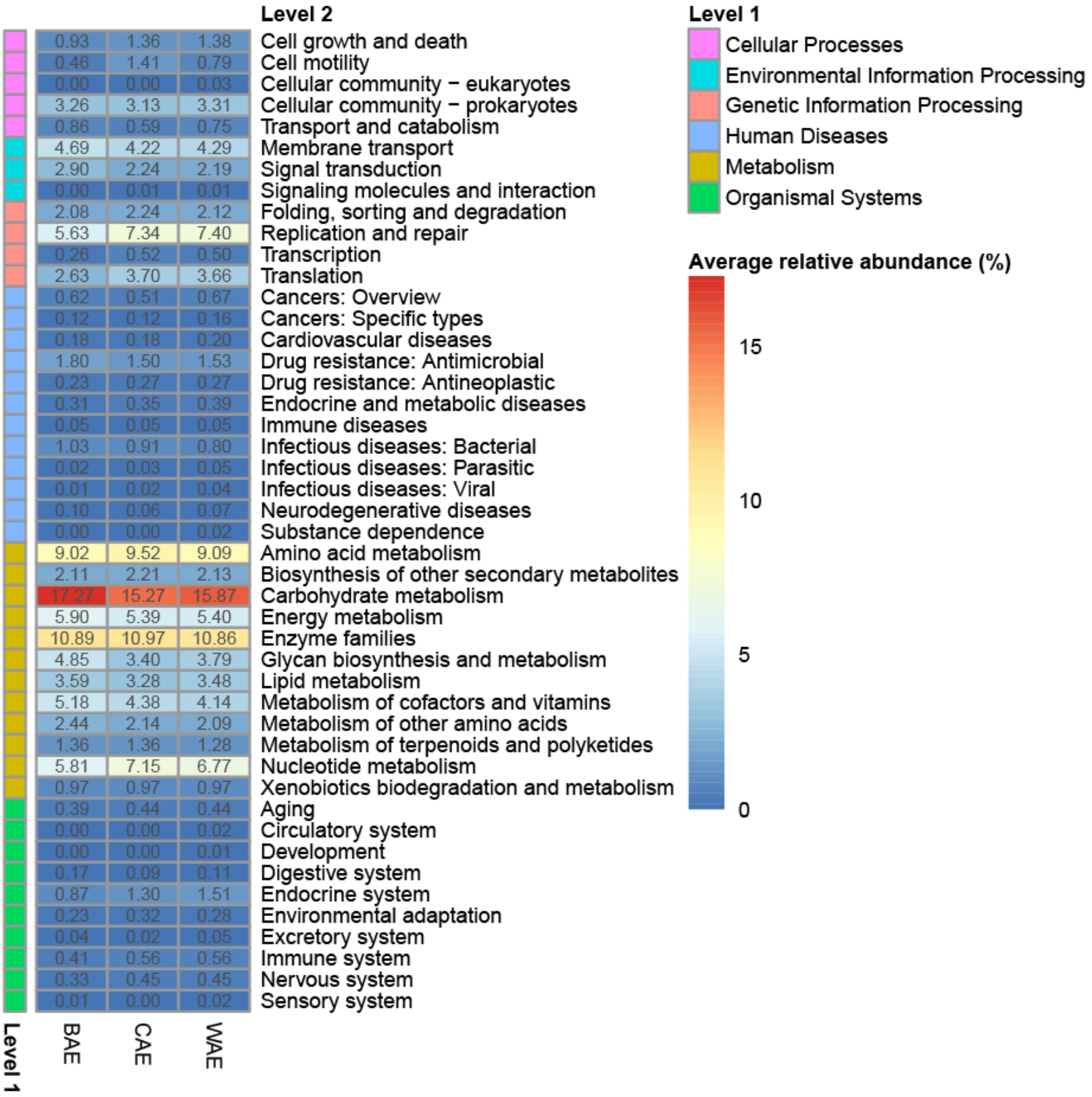

Supplement: Supplementary file 1 [file animals-13-03193-s001.zip › animals-2604646-supplementary materials/Figure S1.pdf]
